# Intracranial solitary fibrous tumor/hemangiopericytoma: Role and choice of postoperative radiotherapy techniques

**DOI:** 10.3389/fonc.2022.994335

**Published:** 2022-09-28

**Authors:** Qiheng Gou, Yuxin Xie, Ping Ai

**Affiliations:** ^1^ Department of Radiation Oncology and Department of Head & Neck Oncology, Cancer Center, West China Hospital, Sichuan University, Chengdu, China; ^2^ Department of Medical Oncology of Cancer Center, West China Hospital, Sichuan University, Chengdu, China; ^3^ Laboratory of Molecular Diagnosis of Cancer, Clinical Research Center for Breast, West China Hospital, Sichuan University, Chengdu, China

**Keywords:** intracranial solitary fibrous tumor/hemangiopericytoma (SFT/HPC), postoperative radiotherapy, intensity-modulated radiotherapy (IMRT), *stereotactic radiosurgery (SRS)*, time to progression

## Abstract

**Background:**

Intracranial solitary fibrous tumor/hemangiopericytoma (SFT/HPC) is a novel rare disease after the 2016 WHO reclassification. Surgery is the main treatment. Postoperative adjuvant radiotherapy is often used, but the effects of different radiotherapy techniques are still unclear. The purpose of this study was to analyze the effects of postoperative radiotherapy (PORT) and different radiotherapy methods on the efficacy of patients with intracranial SFT/HPC.

**Materials and methods:**

We retrospectively analyzed 42 patients with intracranial SFT/HPC who underwent surgical treatment from 2008 to 2022, 20 of whom were treated with postoperative intensity-modulated radiotherapy (IMRT) and 22 with postoperative stereotactic radiosurgery (SRS). The Kaplan–Meier method was used to analyze the disease-free survival (DFS) of all the 42 patients receiving postoperative radiotherapy and the time to progression (TTP) of 22 of these patients experiencing recurrence. A multivariate Cox proportional hazards model was used to detect prognostic factors of survival.

**Results:**

In the analysis of PORT patients, the median DFS was 8.33 years for PORT IMRT patients and 3.04 years for PORT SRS patients. The 10-year DFS incidence was 46.0% in the PORT IMRT group and 27.5% in the SRS group. Among the 22 patients who relapsed, the median TTP of other patients was 1.25 years, of which 3 received radiotherapy alone and 1 received symptomatic treatment, while the median TTP of surgical and surgical combined with radiotheray patients were 1.83 and 2.49 years, respectively (p=0.035).

**Conclusion:**

PORT IMRT could prolong DFS compared with PORT SRS. It indicated that PORT IMRT radiotherapy technology was a feasible option for SFT/HPC. Moreover, TTP results of relapsed patients showed that, surgery and surgery combined with radiotherapy treatments have no significant difference on TTP in relapsed patients, but both of them were better than other treatments.

## Introduction

Hemangiopericytoma (HPC) and solitary fibrous tumor (SFT) are rare primary intracranial tumors. HPC accounts for only 1% of all intracranial tumors (1, 2). Approximately 15% of SFTs show malignant biological behavior, and the others show benign and mesenchymal tumors (3). Recently, researchers found that the Nab2 and STAT6 genes formed a new fusion gene in SFT and HPC samples. This discovery combined the two tumors into a new category, which was named SFT/HPC in the central nervous system (CNS) classification of the World Health Organization (WHO) in 2016 (4). Although several reports have been published describing the pathological characteristics, clinical characteristics and survival outcomes of SFT/HPC (5–8), there is still a lack of sufficient clinical data for this newly defined SFT/HPC.

In addition, previous studies mainly discussed the treatment strategy and prognosis of HPC. Some researchers believe that for HPC, complete surgical resection followed by postoperative radiotherapy (PORT) to the bed is the best strategy (9, 10). Others believed that external irradiation of the tumor bed after surgery appeared to delay recurrence (11).Moreover, PORT was considered the main treatment for HPC. However, a previous study showed that PORT did not improve the local control and survival of HPC. The local control rates after intensity-modulated radiotherapy (IMRT) and stereotactic radiosurgery (SRS) were similar, even though the biological dose of IMRT was much higher than that of SRS (12).

In general, the new classification lacks reliable clinical data, especially leading to doctors’ confusion about treatment strategies. We analyzed the clinical features of 42 patients with SFT/HPC who underwent surgical resection and PORT in our institution. The purpose of this study was to analyze the therapeutic effects of different PORT techniques (IMRT vs. SRS) on 42 patients with SFT/HPC.

## Methods

### Patients and diagnosis

A single-institution, retrospective analysis was performed. We conducted a chart review of primary intracranial malignant tumor cases between January 2008 and December 2022 at West China Hospital, Sichuan University, China. The patients selected for this study should be histologically proven to have primary intracranial SFT/HPC who had surgery as the first therapy. We collected complete clinical data on these patients, including medical history, imaging, pathology, treatment and follow-up. Our study was approved by the Clinical Test and Biomedical Ethics Committee at West China Hospital, Sichuan University (reference number 2022-788). Consent forms were obtained from all participants.

### Treatment

We retrospectively evaluated 42 patients with SFT/HPC treated with PORT by means of IMRT or SRS. Surgical resection methods include gross tumor resection (GTR) and subtotal tumor resection (STR). The treatment after surgical resection included IMRT and SRS. Moreover, disease recurrence occurred in 22 of these patients. Subsequently, different treatment methods were adopted in these 22 patients, including surgery alone, surgery combined with radiotherapy and others. There were only 4 patients with other treatment methods, of whom 3 cases received radiation alone and 1 case received symptomatic treatment. The symptomatic treatment included analgesia, intravenous infusion of 20% mannitol and glucocorticoids.

Twenty patients received fractionated IMRT after surgery with a median prescription dose of 60 Gy (range 40-63 Gy). IMRT is provided with 6 MV photons by a linear accelerator. The clinical target volume (CTV) was defined as the tumor cavity or residual mass plus 1-2 cm margin. An additional 3-5 mm was added to the CTV to plan the target volume (PTV). Twenty-two patients were treated with SRS gamma knife. The single dose at the tumor margin was 11-15 Gy, and the equal dose curve was 40%-50%. In this study, the choice mainly depended on the joint decision of the attending physician and patients.

### Statistics

The characteristics of patients were described by descriptive statistics. Kaplan–Meier analysis was used to estimate OS, and the log rank test was used to evaluate the difference between groups. All statistical variables in univariate analysis were analyzed by the Cox proportional hazards model. OS was defined as the time from diagnosis to patient death. If less than 0.05, the p value was considered significant. All statistical analyses were performed using SPSS version 25.0 (IBM, USA).

## Result

### Participants

Data and clinical follow-up data were collected from a total of 42 patients with intracranial SFT/HPC diagnosed by PORT (**Table 1**). There were 25 men and 17 women. Supratentorial lesions accounted for 71.43%. There were 22 cases of pathological grade II (52.38%) and 20 cases of grade III (47.62%). Gross tumor resection (GTR) was performed in 22 cases (52.38%), and subtotal tumor resection (STR) was performed in 15 cases (35.71%). In **Table 1**, among these patients, a total of 22 patients achieved GTR, of whom 13 patients (56.52%) adopted PORT IMRT and 9 (47.37%) adopted PORT SRS. Among the STR group, 7 patients (30.43%) adopted PORT IMRT and 8 (42.11%) adopted PORT SRS. There were 5 patients with unknown resection types, including 3 patients with PORT IMRT (13.04%) and 2 patients with PORT SRS (10.53). There was no significant difference between the PORT groups.

**Table 1:** Characteristics of patients with SFT/HPC receiving postoperative radiotherapy divided by radiotherapy technique.

	All(n=42)	IMRT(n=20)	SRS(n=22)	*P*
	No. (%)	No. (%)	No. (%)	
Age at diagnosis (years)				0.976
≤ 40	20 (47.62)	11 (47.83)	9 (47.37)	
> 40	22 (52.38)	12 (52.17)	10 (52.63)	
Sex				0.408
Male	25 (59.52)	15 (65.22)	10 (52.63)	
Female	17 (40.48)	8 (34.78)	9 (47.37)	
Pathology grade				0.976
II III	22 (52.38) 20 (47.62)	12 (52.17) 11 (47.83)	10 (52.63) 9 (47.37)	
Intracranial location				0.211
Supratentorial	30 (71.43)	19 (82.61)	11 (57.89)	
Infratentorial	6 (14.29)	2 (8.7)	4 (21.05)	
Both	6 (14.29)	2 (8.7)	4 (21.05)	
Extent of resection				0.629
GTR	22 (52.38)	13 (56.52)	9 (47.37)	
STR	15 (35.71)	7 (30.43)	8 (42.11)	
Unknown	5 (11.90)	3 (13.04)	2 (10.53)	
Recurrence times				< 0.001*
0	20 (47.62)	18 (78.26)	2 (10.53)	
1	13 (30.95)	3 (13.04)	10 (52.63)	
2	4 (9.52)	1 (4.35)	3 (15.79)	
≥ 3	5 (11.90)	1 (4.35)	4 (21.05)	
Liver metastasis				0.265
No	41 (97.62)	23 (100)	18 (94.74)	
Yes	1 (2.38)	0 (0)	1 (5.26)	
Bone metastasis				0.667
No	39 (92.86)	21 (91.3)	18 (94.74)	
Yes	3 (7.14)	2 (8.7)	1 (5.26)	
Other metastasis				0.890
No	40 (95.24)	22 (95.65)	18 (94.74)	
Yes	2 (4.76)	1 (4.35)	1 (5.26)	

IMRT, intensity-modulated radiotherapy; SRS, stereotactic radiosurgery; GTR, gross total resection; STR, subtotal resection.

*P values are statistically significant.

Patients with SFT/HPC tend to relapse. Among them, 22 patients experienced relapse. Thirteen cases (30.95%) recurred once, 4 cases (9.52%) recurred twice, and 5 cases (11.90%) recurred more than three times. In contrast, SFT/HPC is not prone to distant metastasis. Only 1 patient had liver metastasis, and 3 patients had bone metastasis. All 42 patients accepted radiotherapy; among them, 20 patients used IMRT alone, and 22 patients used SRS alone.

The median follow-up time of all patients was 96 months. The difference between the two groups was related to the recurrence times. In addition, compared with the SRS group, the recurrence frequency of IMRT patients after PORT was significantly reduced (p<0.001) (**Table 1**). There was no significant difference in other clinical characteristics (including tumor resection range and tumor grade) between the PORT-IMRT group and the PORT-SRS group.

### Histological findings

**Figure 1:**
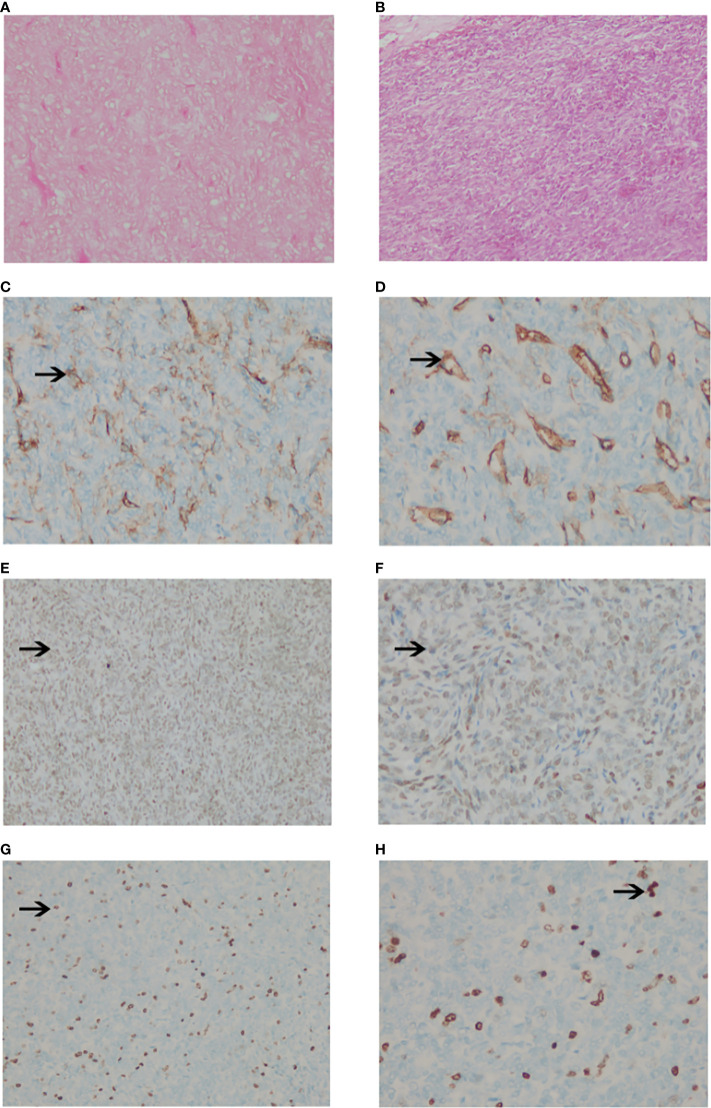
Pathological images of intracranial SPF/HPC. **(A, B)**. The tumor showed compact and uniform cells with a large number of small vascular cavities and compact reticular fibers with hematoxylin and eosin stain HE×100 **(A)** and ×200 **(B)**. **(C, D)**. The positive staining of CD34 was brown granular in the cytoplasm (×100) **(C)** and (×200) **(D)**. **(E, F)**. The positive staining of STAT6 was brown granular in the cytoplasm (×100) **(E)** and (x200) **(F)**. **(G, H)**. Negative staining of Ki67 in tumor cells (×100) **(G)** and (×200) **(H)**.

The pathological diagnosis of SFT/HPC largely depended on the immunohistochemical (IHC) staining results of CD99, CD34, Ki-67 and STAT6. STAT6 positive status is the basis of the new SFT/HPC classification. We performed STAT6 staining again in all patients who were diagnosed with HPC or SPF and underwent surgical resection and PORT. Therefore, all 42 patients included in our study confirmed STAT6 expression by IHC. After applying the 2016 WHO classification, they were reclassified as follows: 22 WHO grade II SFT/HPC patients and 20 WHO grade III SFT/HPC patients. The pathological specimens of all 42 patients were stained with hematoxylin & eosin (H&E), showing extensive vascularization and cellular tumors. The tumor cells were dense and uniform, with a large number of small vascular cavities and dense reticular fibers. The nuclear division, cell morphological heterogeneity and Ki67 percentage of high-grade HPC were more prominent (**Figure 1**).

### Outcome data

**Figure 2:**
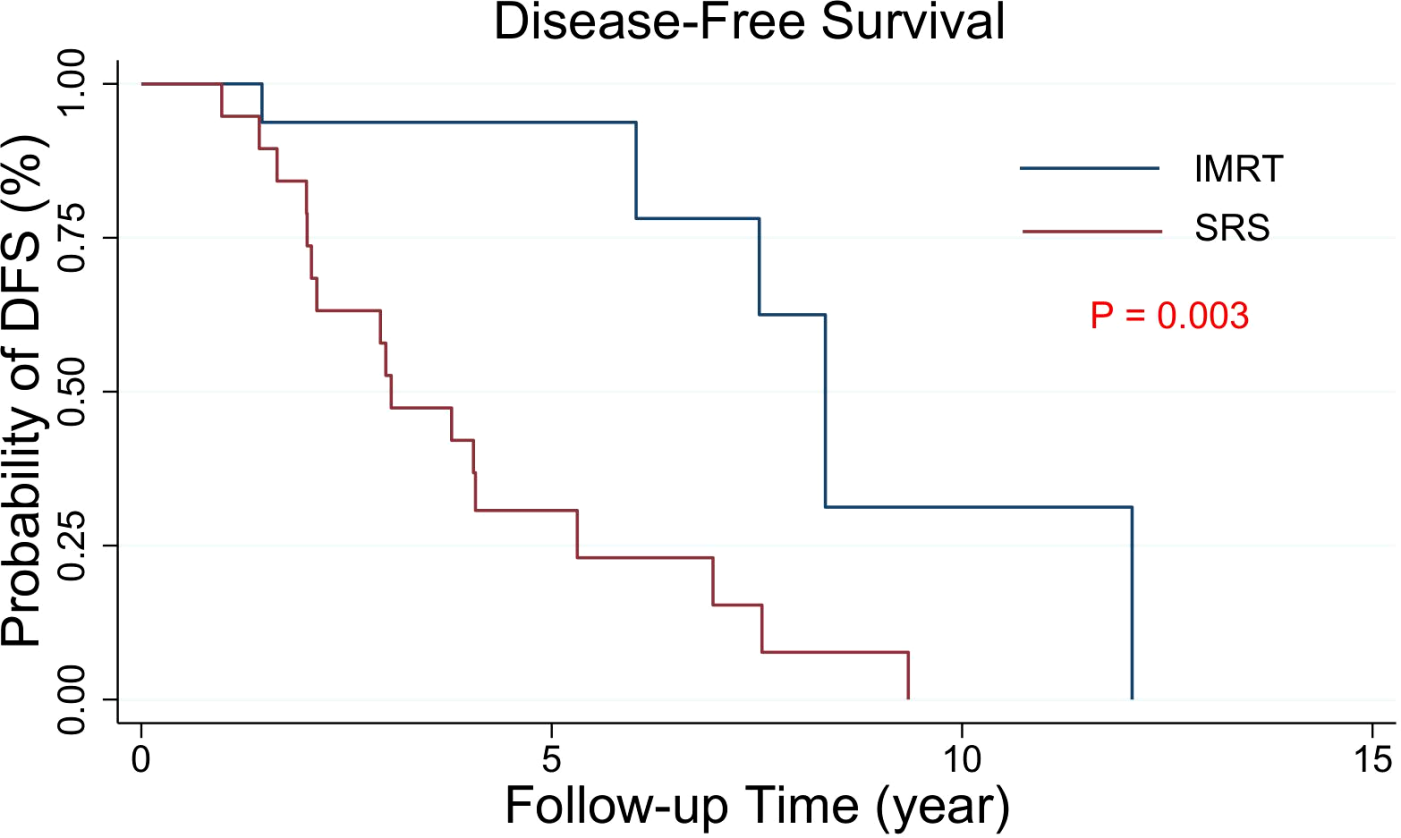
Kaplan−Meier estimates of the DFS curves in intracranial SFT/HPC patients with PORT. The DFS of the two groups with different radiotherapy techniques was significantly different (p = 0.003).

The median DFS was 8.33 years for PORT IMRT patients and 3.04 years for PORT SRS patients. The 10-year DFS incidence was 46.0% in the PORT IMRT group and 27.5% in the SRS group. The results showed that DFS in patients with PORT IMRT was better than that in patients with PORT SRSs (**Figure 2**).

**Figure 3:**
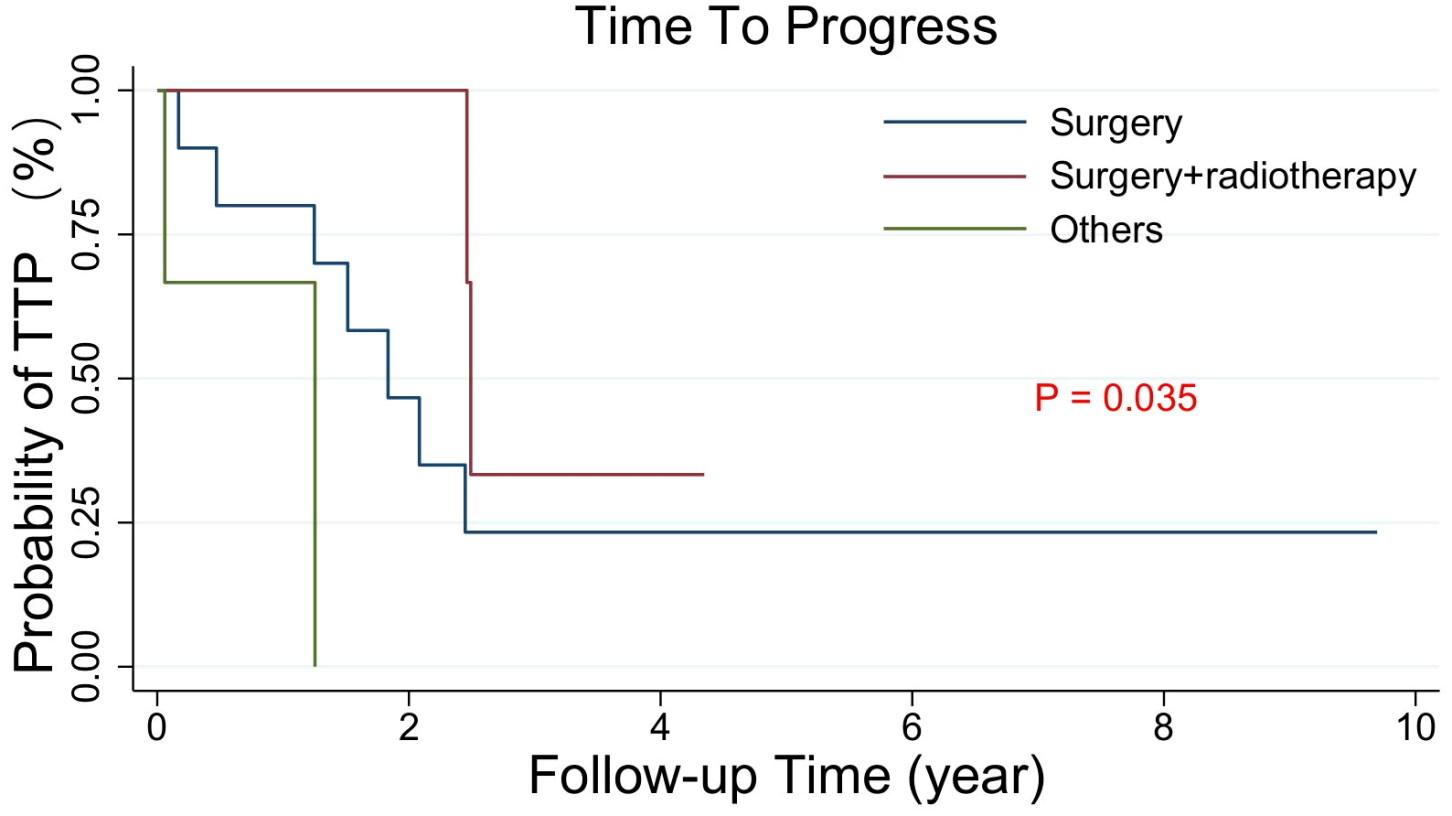
Kaplan−Meier estimated the TTP curves of 22 intracranial SFT/HPC patients with relapse after PORT who received different treatments again (Ssurgery, surgery plus radiotherapy, others). The others included 3 patients who received radiotherapy alone and 1 patient who received symptomatic treatment. The TTP of the surgery and surgery plus radiotherapy groups was similar, but different from that of the other groups (p = 0.003).

Among the 22 patients who relapsed, the median TTP of other patients was 1.25 years, of which 3 received radiotherapy alone and 1 received symptomatic treatment, while the median TTP of surgical patients and surgical combined with radiotherapy patients were 1.83 and 2.49 years, respectively (p=0.035). (**Figure 3**)

### Main results

All 42 patients received surgery and postoperative radiotherapy. Among them, 22 patients had relapse, of whom 10 received simple surgery, 8 received surgery combined with radiotherapy again, 3 received simple radiotherapy, and 1 received symptomatic treatment such as mannitol and glucocorticoids, as shown in **Table 2**.

**Table 2:** COX regression analysis of factors associated with disease-free survival in patients with SFT/HPC receiving post-operative radiotherapy.

Variable	N of patient	N of events	Disease-Free Survival HR (95% CI) p	
Age	42	22	1.00 (0.96–1.06)	0.791
Treatment technique				
IMRT	20	5	1.00	
SRS	22	17	14.17 (1.78–112.63)	0.012*
Sex				
Male	25	12	1.00	
Female	17	10	0.24 (0.08–0.79)	0.019*
Tumor location				
Supratentorial	30	13	1.00	
Infratentorial	6	5	0.75 (0.19–2.96)	0.641
Both	6	4	0.61 (0.16–3.17)	0.647
Pathology grade				
II III	22 20	12 10	1.00 0.40 (0.11–1.52)	0.180
Recurrence times				
1	13	13	1.00	
2	4	4	2.52 (0.57–11.07)	0.729
≥ 3	5	5	1.10 (0.27–4.46)	0.896

N, number; HR, hazard ratio; CI, confidence interval; IMRT, intensity-modulated radiotherapy; SRS, stereotactic radiosurgery.

*P values are statistically significant.

In Cox regression analysis, factors related to DFS in SFT/HPC patients receiving postoperative radiotherapy showed that PORT IMRT obtained better DFS than PORT SRS (HR 14.17[1.78-112.63], p=0.012). On the other hand, sex is a factor influencing DFS. We found that DFS was longer in women than in men (HR 0.24[0.08-0.79], p=0.019). Furthermore, tumor location, pathological grade and recurrence time had no significant relationship with DFS.

Moreover, among the 22 patients who relapsed, Cox regression analysed factors related to progression time in patients with recurrent SFT/HPC. We found no significant correlation with progression time (including age, sex, tumor location and pathological grade). The TTP results of surgery or surgery combined with radiotherapy were better than those of other treatments, including radiotherapy alone and symptomatic treatment (HR 10.93 [1.01–117.91], p=0.049).

Although the median TTP time of surgery combined with radiotherapy was not significantly different from that of surgery alone (HR 0.18 [0.02–1.64], p=0.129), the median TTP time of surgery combined with radiotherapy was longer than that of surgery alone (2.49 vs.1.83 years) (**Table 3**). However, there were limited number of patients in each group, and further validation is needed.

**Table 3:** COX regression analysis of factors associated with time to progress in patients with recurrent SFT/HPC.

Variable	N of patient	N of events	Disease-Free Survival HR (95% CI)p	
Age	22	11	1.00 (0.90–1.12)	0.897
Treatment mode				
Surgery	10	7	1.00	
Surgery+radiotherapy	8	2	0.18 (0.02–1.64)	0.129
Others	4	2	10.93 (1.01–117.91)	0.049*
Sex				
Male	12	7	1.00	
Female	10	4	0.27 (0.05–1.47)	0.130
Tumor location				
Supratentorial	13	6	1.00	
Infratentorial	5	3	1.24 (0.15–10.08)	0.838
Both	4	2	0.74 (0.07–7.48)	0.796
Pathology grade				
II III	12 10	6 5	1.00 0.66 (0.12–3.71)	0.635

Others contain 3 cases received radiotherapy alone and 1 case received symptomatic treatment.

N, number; HR, hazard ratio; CI, confidence interval.

*P values are statistically significant.

## Discussion

SFT/HPC is a rare disease that is also very rare in the literature because no study has a large number of cases and satisfactory follow-up time. Some studies revealed that the range of tumor resection was crucial. Complete tumor resection plays a key role in local control and survival (13–15). There is no relevant recommendation on the choice of PORT technology. The choice mainly depends on the joint decision of the attending physician and patients in our study. The results showed that PORT IMRT was helpful to prolong DFS in patients compared with PORT SRS. In our study, surgical resection and PORT were both superior to other types of treatment strategies.

Brunori et al. found that, unlike meningiomas, the incidence rate of SFT/HPC was higher in men than in women (16). A meta-analysis of 523 patients showed that the incidence rate in male patients was higher than that in female patients under the age of 45, while the trend was the opposite for patients aged 45 and above (17). In our study, there was no significant difference between groups in age or sex at baseline.

In a previous study that included 191 patients, male patients with HPC had a recurrence risk more than 8 times that of female patients (18), while Damodaran et al. showed that male patients had a higher survival rate (19). Our study found that compared with female patients, men had worse DFS (disease-free survival HR (95% CI) 0.24 (0.08 – 0.79), P = 0.019), which may be more related to the recurrence risk of male patients, but it is worth further investigation.

Since the World Health Organization merged SFT and HPC into a new disease called SFT/HPC in 2016, relevant research has been very limited. Furthermore, most of the previous studies on the role of PORT in intracranial HPC are based on single center analysis. The number of patients is limited, and the results are often inconsistent. Meta-analysis and research based on cumulative databases have been carried out to overcome the problems of small case series, but the results are also inconsistent (15, 20, 21). Although the role of PORT in patients with SFT/HPC is unclear, the general consensus is that PORT is beneficial to patients undergoing surgery.

Some studies have found that compared with STR alone, PORT following STR can improve overall survival (OS) and recurrence-free survival (RFS) (20, 22, 23). Other studies have reported that PORT following GTR can also prolong OS (1, 10, 21, 24) or improve local control (25, 26). In contrast to the above, some authors report that PORT after GTR has no effect on survival (20, 27) or should not be used except for recurrent patients (28, 29). In our study, we found that different PORT strategies had a significant impact on disease-free survival. The median DFS of patients receiving PORT IMRT was 8.33 years, which was significantly different from that of patients receiving PORT SRS, which was 3.04 years (p=0.03). Compared with surgery, multivariate analysis showed that postoperative radiotherapy had no predictive effect on survival, but compared with other treatments, PORT and surgery were beneficial to DFS.

SRS has been used in patients with residual or recurrent intracranial HPC (9, 28, 30–34). It was reported that postoperative SRS can better control local tumors in patients with intracranial HPC (9). However, in our analysis, patients with SFT/HPC with SRS had poorer DFS than patients receiving IMRT. The tumor biological effective dose (BED) of the SRS and IMRT groups of SFT/HPC with an α/β ratio of 10 Gy was 33.6-41.6 Gy_10_ and 72 Gy_10_, respectively. The biological effect of IMRT is much higher than that of SRS, which may lead to higher local control. This might be due to the higher BED inducing a greater local effect. There is no consensus on the optimal IMRT radiation dose for intracranial SFT/HPC. Some centers reported that the local control rate was improved when the tumor margin dose was not less than 60 Gy (26). In addition, the SRS radiotherapy plan mainly irradiates the tumor bed, while the IMRT plan irradiates the surrounding 1-2 cm CTV area in addition to the tumor bed. This difference in concept may lead to radiotherapy being superior to SRS in local control and recurrence prevention.

According to our analysis, PORT IMRT compared with PORT SRS seems to improve DFS. However, due to the limitations of this study, including the small sample size and its retrospective nature, caution should be taken in interpreting the current research results. To study the exact effect of PORT on local control and survival and the different effects between IMRT and SRS as PORT options, a multicenter randomized trial with a larger sample size is needed.

## Conclusion

In this retrospective analysis, the DFS of PORT IMRT might be longer than that of SRS. This indicated that PORT IMRT radiotherapy technology was a feasible option. Moreover, TTP results of relapsed patients showed that, surgery and surgery combined with radiotherapy treatments have no significant difference on TTP in relapsed patients, but both of them were better than other treatments.

## Data availability statement

The original contributions presented in the study are included in the article/**Supplementary Material**. Further inquiries can be directed to the corresponding author.

## Ethics statement

The studies involving human participants were reviewed and approved by Clinical Test and Biomedical Ethics Committee at West China Hospital, Sichuan University. The ethics committee waived the requirement of written informed consent for participation.

## Author contributions

Conceptualization: PA. Data curation: QG and YX. Project administration: PA. Supervision: PA. Writing-original draft: QG. Revision: QG and YX. All authors made a significant contribution to the work reported, gave final approval of the version to be published; have agreed on the journal to which the article has been submitted; and agree to be accountable for all aspects of the work.

## Funding

This work was supported by National Natural Science Foundation of China (81902723), and 1·3·5 project for disciplines of excellence–Clinical Research Incubation Project, West China Hospital, Sichuan University (2021HXFH050).

## Conflict of interest

The authors declare that the research was conducted in the absence of any commercial or financial relationships that could be construed as a potential conflict of interest.

## Publisher’s note

All claims expressed in this article are solely those of the authors and do not necessarily represent those of their affiliated organizations, or those of the publisher, the editors and the reviewers. Any product that may be evaluated in this article, or claim that may be made by its manufacturer, is not guaranteed or endorsed by the publisher.

## Abbreviations

SFT, solitary fibrous tumor; HPC, hemangiopericytoma; DFS, disease-free survival; TTP, time to progression; PORT, postoperative radiotherapy; GTR, gross tumor resection; STR, subtotal tumor resection; CTV, clinical target volume; BED, biological effective dose. WHO, World Health Organization; IMRT, intensity-modulated radiotherapy; SRS, stereotactic radiosurgery.
