# Supplementary material for: Intracranial solitary fibrous tumor/hemangiopericytoma: Role and choice of postoperative radiotherapy techniques
Source: Front Oncol. 2022 Sep 28;12:994335. doi: 10.3389/fonc.2022.994335 (PMC9554559; doi:10.3389/fonc.2022.994335)
Supplement: Supplementary file 1 [file Table_1.docx]

**Table S1: COX regression analysis of factors associated with time to progress in patients with recurrent SFT/HPC.**

| Variable | N of  patient | N of  events | Disease-Free Survival  HR (95% CI) ^†^ p | |
| --- | --- | --- | --- | --- |
| Treatment mode |  |  |  |  |
| Surgery+radiotherapy | 8 | 2 | 1.00 |  |
| Surgery | 10 | 7 | 5.48 (0.61–49.41) | 0.129 |
| Others | 4 | 2 | 59.9 (1.63–219.33) | 0.026* |

Note: Others contain 3 cases received radiotherapy alone and 1 case received symptomatic treatment.

Abbreviations: N, number; HR, hazard ratio; CI, confidence interval.

^†^ HRs were adjusted for age at diagnosis, sex (male or female), tumor location (supratentorial, infratentorial, or both), pathology grade (II or III).

*P values are statistically significant.
